# Evaluation of the Biological Activity of Glucosinolates and Their Enzymolysis Products Obtained from *Lepidium meyenii* Walp. (Maca)

**DOI:** 10.3390/ijms232314756

**Published:** 2022-11-25

**Authors:** Suitong Yan, Jinchao Wei, Rui Chen

**Affiliations:** 1 College of Chemistry and Chemical Engineering, Yunnan Normal University, Kunming 650500, China; 2 Institute of Chemistry, The Chinese Academy of Sciences, Beijing 100190, China

**Keywords:** *Lepidium meyenii* (Maca), glucosinolate, enzymolysis product, biological activity

## Abstract

Glucosinolates (GLS) were extracted and purified from *Lepidium meyenii* (Maca) root. Purified GLS were analyzed without desulfation by UPLC–ESI–MS. Glucosinolates were decomposed into benzyl isothiocyanate (BITC) by thioglucosidase. DPPH radical scavenging activity, ABTS radical scavenging activity, and reducing power were used to evaluate antioxidant activity of Maca crude extract (MCE), total GLS, and BITC. Maca crude extract showed the highest antioxidant activity among them, and BITC showed no antioxidant activity at concentrations less than 10 mg/mL. Cytotoxicity on five human cancer cell lines and the inhibition rate of NO production were used to evaluate the activity of anti-cancer and anti-inflammatory of total GLS and BITC. The inhibition rate of NO production of 50 μg/mL BITC can reach 99.26% and the cell viability of 100 μg/mL BITC on five tumor cell lines is less than 3%. The results show that BITC may be used as a promising anti-cancer and anti-inflammatory drug.

## 1. Introduction

*Lepidium meyenii* Walp. (Maca), a member of an annual or biennial herb of Brassicaceae family, is cultivated above an altitude of 3500 m in Peru. This plant can well adapt to the living environment of high altitude, strong ultraviolet radiation, low oxygen level, and changeable climate. Maca has been widely planted in China after it was successfully naturalized in Lijiang, Yunnan in 2002. The edible part of Maca is commonly known as root [1].

There are a variety of primary metabolites, secondary metabolites, and minerals in Maca. The secondary metabolites, including alkaloids, macaenes, glucosinolates (GLS), sterols, polyphenols, and polysaccharides, are considered as the main components with potential pharmacological value in Maca root. These components make Maca have a series of biological activities, such as improvement of reproductive health, antioxidation, antifatigue, anti-cancer, antiosteoporosis, hepatoprotection, immunoregulation, and neuroprotection [2]. Based on its pharmacological value, the health food associated with Maca has been welcomed by consumers in the market [3]. However, evaluation of the bioactivity of secondary metabolite in Maca is still one of the hot topics in the study of its components.

GLS are one of the important secondary metabolites in cruciferous plant. In 1970, after analyzing the crystalline of GLS by X-ray, Marsh and his coauthor considered that GLS were made up of three parts including a thio-β-D-glucopyranoside chain, a sulfonate group, and a side chain (R group) [3,4,5]. Hence, GLS were also divided into aliphatic glucosinolate, aromatic glucosinolate, and indole glucosinolate according to the type of R group [4,5].

It was reported that Maca contained nine kinds of GLS, among which the aromatic GLS mainly included benzyl glucosinolate (GTL), *m*-methoxybenzyl glucosinolate (GLH), and *p*-hydroxybenzyl glucosinolate (GSB). The total content of GTL, GLH, and GSB arrived at 99% in total GLS content [6]. There was a difference of total GLS content in different parts of Maca. Maca seed contained the highest total GLS content, followed by fresh hypocotyl, dry hypocotyl, flour, and fresh leaf, successively [7].

When plant tissues are damaged, myrosinase stored in myrosin cells will be released to hydrolyze GLS and to release several products, such as epithionitriles, isothiocyanates (ITCs), nitriles, oxazolidine-2-thiones, and thiocyanates [8]. GLS and their enzymolysis products were known for cancer chemoprotective activity with potential antiproliferative, apoptosis-promoting, and redox regulatory activities [8,9,10,11,12,13,14]. Cytotoxicity of benzyl isothiocyanate (BITC) was evaluated through tests in vivo and in vitro [15,16,17]. It indicated that BITC had inhibitory effect on breast cancer stem cells [17]. In addition, BITC, allyl isothiocyanate (AITC), and phenethyl isothiocyanate (PEITC) showed the significant inhibition effects on lung cancer and esophageal cancer in rat models and ITCs were considered with anti-cancer activity [18,19,20,21].

We know of no report on antioxidation of GLS and their enzymolysis products and anti-cancer activity of GLS in Maca. In this paper, antioxidation of total GLS and their enzymolysis product BITC was firstly evaluated based on the DPPH radical scavenging capacity, ABTS radical scavenging ability, and reducing power. Then, cytotoxicity of total GLS and BITC was evaluated through the inhibition rate of five human tumor cells including breast cancer MCF-7, colon cancer SW480, leukemia HL-60, liver cancer SMMC-7721, and lung cancer A549. Last, their anti-inflammatory activity was evaluated by Griess method through detection of nitrite (NO_2_^−^). The results show that antioxidation of total GLS and BITC is not correlated with their anti-cancer and anti-inflammatory activities.

## 2. Results and Discussion

### 2.1. Analysis of GLS

UV-Vis analysis: Since total GLS were a kind of colorless compound, they were scanned by UV-Vis at the wavelength range of 190–400 nm. There is a wide absorption band at 190–280 nm, and λ_max_ is located at 229 nm, which is the characteristic absorption band of S–C≡N in the UV region (Figure S1 in Supplementary Materials). Hence, 229 nm was selected as the detection wavelength of ultra-high performance liquid chromatography (UPLC).

FT-IR analysis: In FT-IR of GLS (Figure S2 in Supplementary Materials), the band at 3347.47 cm^−1^ corresponds to O–H stretching vibration. Because there is methylene on the side chain and sugar group of glucosinolate, it can be judged that the band at 2929.80 cm^−1^ corresponds to C–H stretching vibration of methylene. The bands at 1667.34 cm^−1^, 1401.23 cm^−1^, and 1046.12 cm^−1^ correspond to C=N, C–O–C, and S=O stretching vibration, respectively. This result is consistent with Yuan’s report [22].

UPLC–ESI–MS analysis: GLS have poor retention effect on the reversed-phase C_18_ column due to its strong polarity. Hence, it is necessary to reduce the polarity of GLS to detect GLS by conventional method. Desulfation is one of the usual techniques to reduce the polarity and can form desulfurized GLS. In the view of evaluation of biological activity of GLS, a UPLC–ESI–MS was established to analyze the complete structure of GLS from Maca.

**Figure 1:**
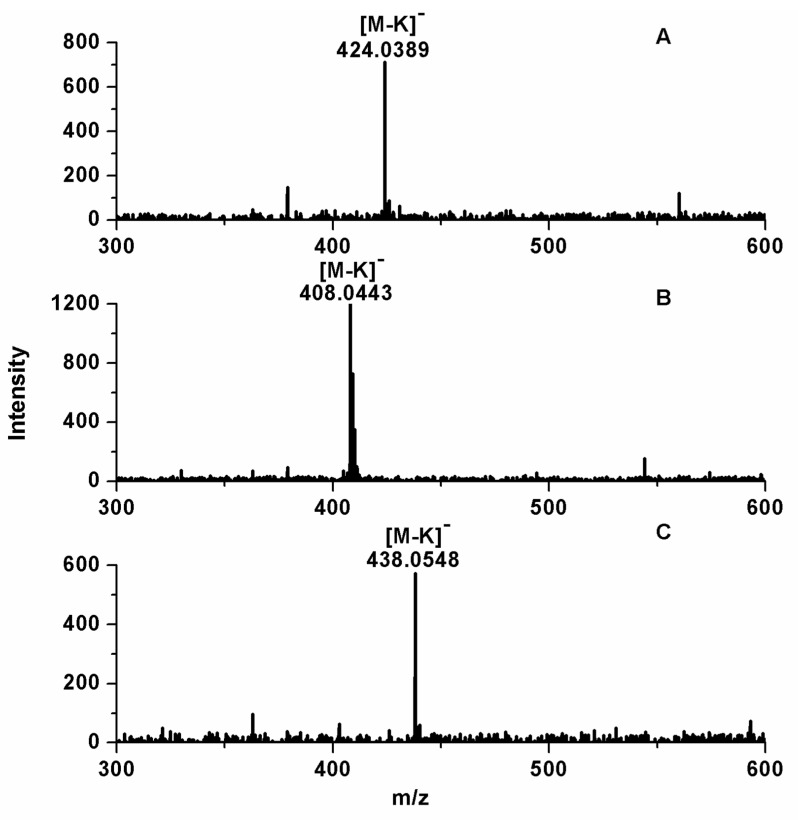
Negative ion electrospray ionization-mass spectra of *p*-hydroxybenzyl glucosinolate (**A**), benzyl glucosinolate (**B**), and *m*-methoxybenzyl glucosinolate (**C**) in GLS from Maca, respectively.

Three chromatographic peaks of samples were detected by UPLC within 15 min (Figure S3 in Supplementary Materials). It was found that there was a large difference of retention time between the standards and samples. This difference in retention time may be due to the influence of matrix on samples. Comparing Figure S3A with Figure S3C, better separation and enrichment of samples were obtained through the purification with D301 macroporous anion resin and acidic alumina column. Mixed standards were added to the sample for qualitative analysis in Figure S3D. Compared with Figure S3C, after addition of the standards, only the peak areas increased, but no other peaks appear in Figure S3D. In order to further determine these compounds, these samples were analyzed by MS in negative ion mode and the results are shown in Figure 1 and Table S1. In MS, *m*/*z* of quasi-molecular ions [M-K]^−^ at 424.0389, 408.0443, and 438.0548 correspond to GSB, GTL, and GLH, respectively. The relative errors of *m*/*z* between measured adducts and theoretical adducts are no more than 5 ppm.

There was a remarkable difference in GLS content between fresh Maca and dried Maca in Table 1. For example, GTL content in fresh Maca is 24.31 ± 2.5 mg/g, while that in dried Maca is 19.37 ± 0.67 mg/g. Total GLS content in fresh Maca is 34.12 ± 2.4 mg/g, and that in dried Maca is 26.32 ± 1.4 mg/g. This conclusion was in agreement with the report that GLS content in fresh Maca was greater than that in dried Maca [7]. The fact may be explained that some GLS are hydrolyzed by thioglucosidase or broken down in the drying process [23].

### 2.2. Analysis of Enzymatic Hydrolysis Products

Myrosinase can effectively control the enzymolysis of GLS [24]. Though GLS in fresh Maca showed the inactivity to myrosinase at pretreatment, GLS in Maca can be degraded into ITCs by myrosinase at 40 °C [25].

**Figure 2:**
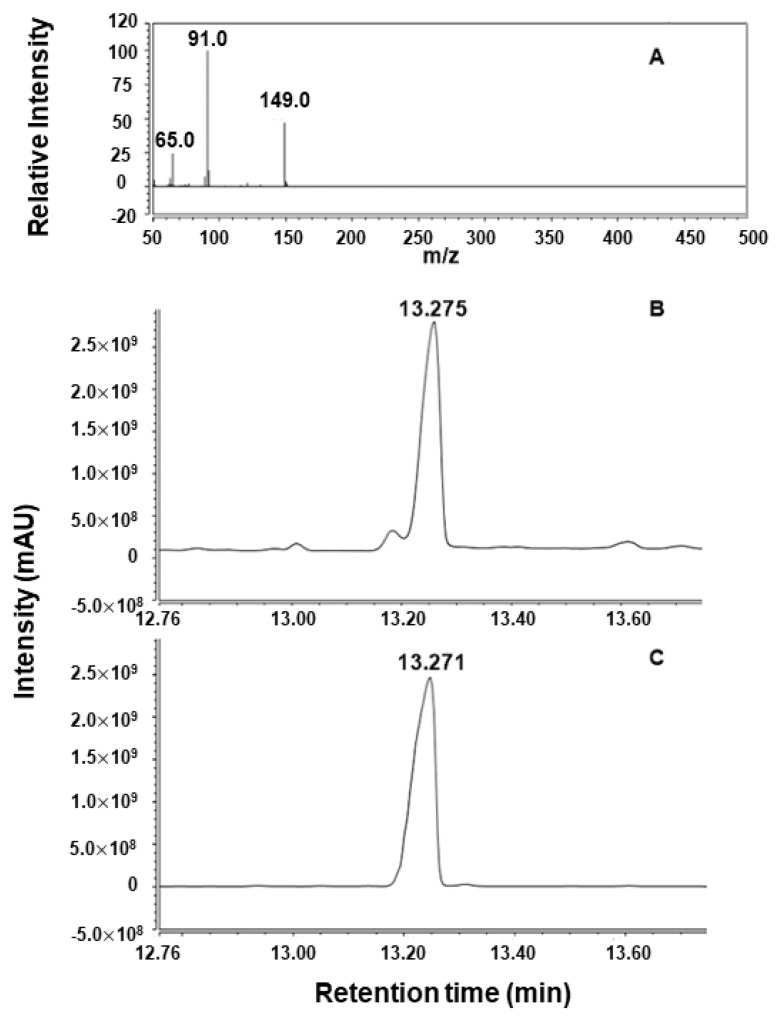
Determination of enzymolysis product from GLS. (**A**) Mass spectrum of enzymolysis product from GLS, (**B**) gas chromatogram of enzymolysis product from GLS, and (**C**) gas chromatogram of benzyl isothiocyanate.

The peak of enzymolysis product of the purified GLS was located at *m*/*z* 91 detected by GC MS, which corresponded to [M-NCS]^+^ (Figure 2A). The retention time of enzymolysis product and BITC was 13.275 min and 13.271 min, respectively (Figure 2B,C). By comparison of retention time, *m*/*z*, and result in NIST standard library, this enzymolysis product was determined to be BITC.

### 2.3. Evaluation of Antioxidation Activity

Comparative analysis method was used to evaluate the antioxidation activity of MCE and total GLS. Standard derivation (SD) was used to evaluate the dispersion degree of experimental data.

**Figure 3:**
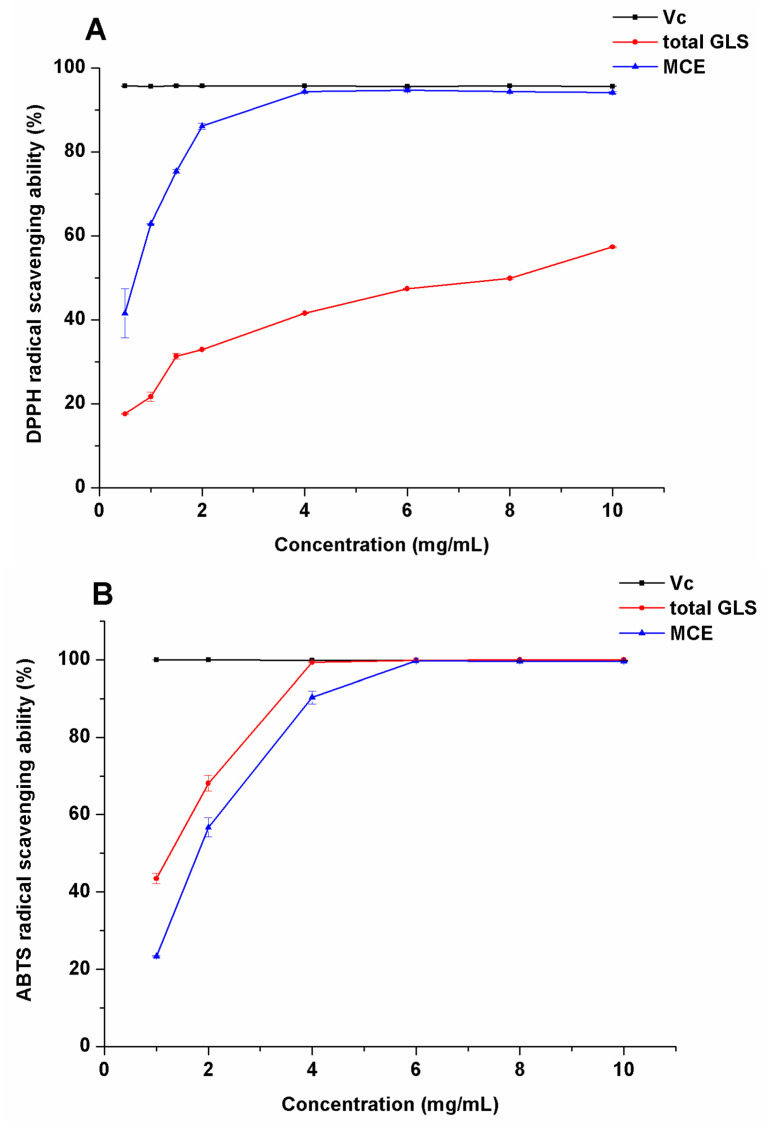

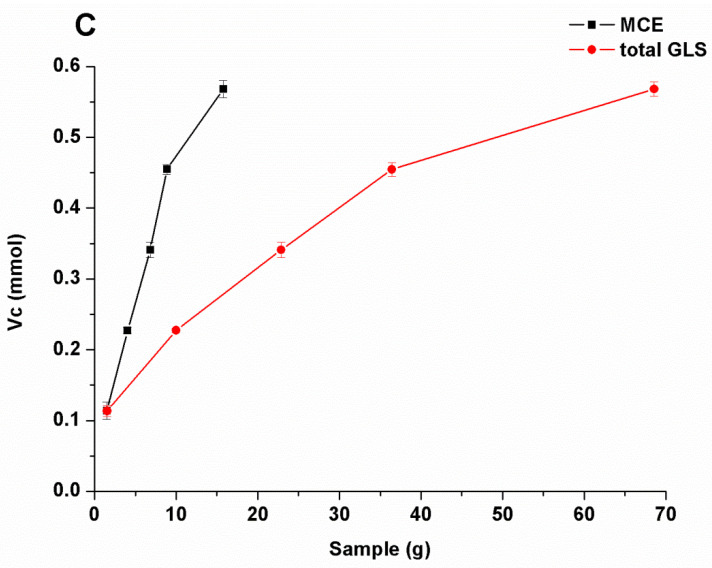
Antioxidation activities of GLS and their enzymolysis product. (**A**) 1,1-diphenyl-2-picrylhydrazyl (DPPH) radical scavenging activity of Maca crude extract and total GLS at 0.5–10 mg/mL, (**B**) 2,2′-azino-bis-(3-ethylbenzothiazolin-6-sulfonic acid) (ABTS) radical scavenging ability of total GLS and Maca crude extract solutions at 0.5–10 mg/mL, and (**C**) the reducing power of Maca crude extract and total glucosinolates.

DPPH radical scavenging capacity: To study the antioxidation ability of total GLS, we compared DPPH radical scavenging ability of MCE with that of total GLS. In Figure 3A, with the increase of the concentration (0.5–10 mg/mL) of MCE and total GLS, DPPH radical scavenging ability increased, showing a positive correlation between concentration and DPPH radical scavenging ability. DPPH radical scavenging rate of MCE observably increased with the increase of concentration (2–4 mg/mL). It tended to increase slowly at concentrations above 4 mg/mL. DPPH radical scavenging rate of 10 mg/mL MCE reached 95.73%, which was near to that of Vc at the same concentration. DPPH radical scavenging capacity of total GLS increased more slowly than that of MCE with the increase of concentration. DPPH radical scavenging capacity of total GLS was 57.37% at 10 mg/mL. The reason that DPPH radical scavenging capacity of total GLS was lower than that of MCE may be attributed to a variety of components such as macamides, polysaccharides, sterols, and flavonoids with antioxidant properties in MCE [26].

ABTS radical scavenging capacity: In Figure 3B, ABTS radical scavenging capacity of total GLS and MCE was positively correlated with their concentrations (0.5–10 mg/mL). Total GLS showed better ABTS radical scavenging capacity than DPPH radical scavenging capacity with concentrations ranging from 1 to 4 mg/mL. ABTS radical scavenging capacity of MCE was 99.35% at 4 mg/mL, while that of total GLS was 90.27% at the same concentration. ABTS radical scavenging capacity of total GLS reached 99.66% at 6 mg/mL, which was equivalent to that of V_C_ and MCE at the same concentration.

Detection of reducing power: The determination of reducing power is a common method to evaluate the antioxidant activity of samples. In this experiment, the reducing power of sample was measured with reference to the method reported by Wang C.C. et al. [27]. In Figure 3C, reducing power of 0.227 mmol Vc was equivalent to that of 4.03 g MCE or 9.99 g total GLS. Hence, reducing power of MCE is higher than that of total GLS. This result of the low antioxidant activity of total GLS is in agreement with a previous finding that GLS showed no evidence for chemical defense signaling in *Brassica rapa* [28].

These three antioxidation assays of BITC were carried out under the same condition. BITC showed no antioxidant activity when concentration of BITC was less than 10 mg/mL.

### 2.4. Anti-Cancer Activity of Extracts, GTL, and BITC

**Figure 4:**
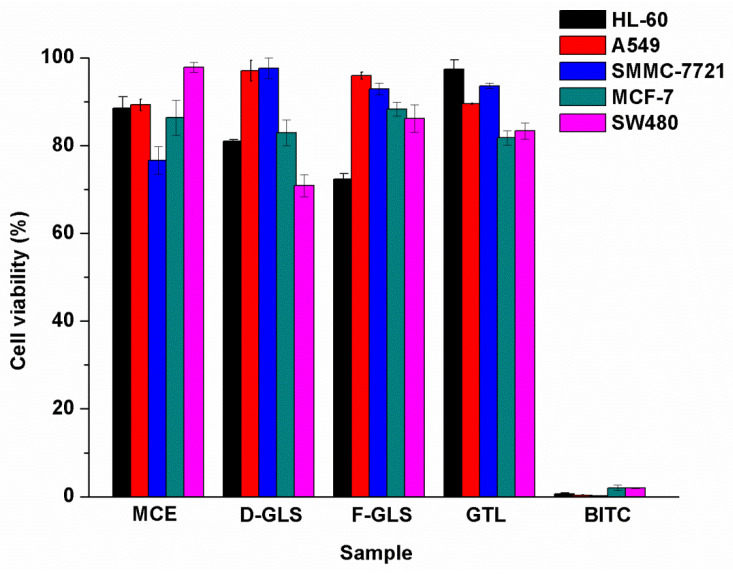
Cytotoxicity in vitro of GLS extracted from dried Maca (D-GLS), GLS extracted from fresh Maca (F-GLS), MCE, benzyl glucosinolate (GTL), and benzyl isothiocyanate (BITC) on five human cancer cells at 100 μg/mL, respectively.

Some references reported that anti-cancer activity of Maca was related to GLS, while others reported that it related to GLS and their enzymolysis products [9,29]. To elucidate this problem, we selected GLS extracted from fresh Maca (F-GLS) and dried Maca (D-GLS), MCE, GTL, and BITC as samples to evaluate their cytotoxicity in vitro on five human cancer cells including leukemia HL-60, lung cancer A549, liver cancer SMMC-7721, breast cancer MCF-7, and colon cancer SW480 in this assay. As seen in Figure 4, there was much difference in the inhibitory activities among F-GLS, D-GLS, MCE, and GTL on the five tumor cell lines. Cytotoxicities of F-GLS, D-GLS, MCE, and GTL on these five human cancer cells were less than 50%, and thus considered to have no anti-cancer activity.

However, 100 μg/mL BITC showed strong inhibitory activities (*p* < 0.05) on five tumor cell lines, and inhibitory rates of BITC on leukemia HL-60, lung cancer A549, liver cancer SMMC-7721, breast cancer MCF-7, and colon cancer SW480 were 99.32 ± 0.19%, 99.67 ± 0.07%, 99.85 ± 0.09%, 97.98 ± 0.64%, and 98.01 ± 0.11%, respectively. It was concluded that although total GLS in Maca had no obvious cytotoxicity on five tumor cell lines, they were an important kind of active intermediate [5,30]. BITC, the enzymolysis product of total GLS, had excellent cytotoxicity on the five tumor cell lines.

The comparative analysis method was used to evaluate the cytotoxicity of BITC on five tumor cell lines. SD was used to evaluate the dispersion degree of experimental data. Compounds with 50% growth inhibition rate were further evaluated at concentrations of 0.16, 0.8, 4, 20, and 100 μg/mL in triplicate, as seen in Table 2 and Table S2. Cell viability of the five tumor cell lines varies with the concentration of BITC. For example, cell viabilities of leukemia HL-60, lung cancer A549, and breast cancer MCF-7 decrease with the concentration of BITC from 0.16 μg/mL to 100 μg/mL. In Table 3, with DDP and paclitaxel as positive controls, the half inhibitory concentration (IC_50_) value of each compound was calculated with Cory’s report [31]. Although cytotoxicity of BITC on five tumor cell lines was lower than that of paclitaxel, it was remarkably higher than that of the antitumor drug DDP. The results show that BITC has cytotoxicity on the five tumor cell lines and can be applied as a promising anti-cancer drug.

### 2.5. Anti-Inflammatory Activity

Many diseases are associated with a considerable inflammatory response, indicative of a host defense mechanism [32]. Inflammation results from the host’s immune response to pathogenic challenges or tissue damage [33]. Under normal conditions, after the response is over, the tissue structure and function will return to normal. When immune cells are stimulated by microbial endotoxin and inflammatory mediators, they will produce a large amount of inducible nitric oxide synthase and NO for immune response. Therefore, inhibition of NO production is considered as a direct indicator of anti-inflammatory activity of sample. In Table 4, anti-inflammatory activity of F-GLS, D-GLS, GTL, and BITC was evaluated at 50 μg/mL. Total GLS and GTL showed no anti-inflammatory activity compared with the positive control, while BITC showed strong anti-inflammatory activity (*p* < 0.05) with a 99.26% inhibition rate of NO. We used the concentration of 50 μg/mL to perform anti-inflammatory assay. In this assay, we further found that 50 μg/mL BITC has toxicity when MTS was added to residual medium to test the cell viability. Then, we adjusted the concentration of BITC to 1.56 μg/mL. We found that 1.56 μg/mL BITC has no toxicity and shows excellent anti-inflammatory response. In fact, 1.56 μg/mL BITC showed a 88.44% inhibition rate of NO. This study shows that anti-inflammatory activity of BITC is better than that of NOSI at the same concentration. This study further shows that the anti-inflammatory drug has anti-cancer activity because chronic inflammation contributes to about one in four of all cancer cases cancer [34].

## 3. Materials and Methods

### 3.1. Materials

Yellow Maca was purchased from Fengxi Food Management Co., LTD. (Kunming, China). This plant was cultivated in Huize, Yunnan. Reference standards including GLH, GSB, and GTL were purchased from Wuhan Costan Technology Co., Ltd. (Wuhan, China) and their purity was greater than or equal to 98%. ABTS and barium acetate were purchased from Shanghai Macklin Biochemical Co., Ltd. (Shanghai, China) and their purity was greater than or equal to 99%. DPPH was purchased from Shanghai Civic Chemical Technology Co., Ltd. (Shanghai, China) and its purity was greater than or equal to 97%. Griess reagent, lipopolysaccharide (LPS), nitric oxide synthase inhibitor (NOSI), and thioglucosidase were purchased from Sigma-Aldrich Co., Ltd. (Shanghai, China). Dimethyl sulfoxide (DMSO) was purchased from Biological Industries (Shanghai, China) and their purity was greater than or equal to 99%. Paclitaxel and DDP were purchased from Meilun Biotech Co., Ltd. (Shanghai, China). Five human tumor cancer cells including breast cancer MCF-7, colon cancer SW480, leukemia HL-60, liver cancer SMMC-7721, and lung cancer A549 were purchased from ATCC (Manassas, VA, USA). RMPI-1640 and DMEM mediums were purchased from Biological Industries (Kibbutz Beit-Haemek, Israel). Mouse monocyte macrophage RAW264.7 was purchased from Shanghai Cell Bank of Chinese Academy of Sciences (Shanghai, China). BITC was purchased from Shanghai Adamas Co., Ltd. (Shanghai, China) and its purity was greater than or equal to 99%. CCl_3_COOH, CF_3_COOH, FeCl_3_, Na_2_HPO_4_·12H_2_O, NaH_2_PO_4_·2H_2_O, K_3_[Fe(CN)_6_], K_2_S_2_O_8_, and Vitamin C (Vc) were purchased from Tianjin Fengchuan Chemical Reagent Technology Co., Ltd. (Tianjin, China) and their purity was greater than or equal to 99%. D301 macroporous anion resin was purchased from Tianjin Jinxi’na Environmental Protection Material Technology Co., Ltd. (Tianjin, China). Acidic alumina column was purchased from Shanghai Macklin Biochemical Co., Ltd. (Shanghai, China).

### 3.2. Extraction and Purification

Before extraction, fresh root of Huize Maca was heated and enzymes were denatured at 100 °C for 15 min, then dried in vacuum at 60 °C for 6 h [35]. Maca was grinded by grinder for no more than 5 min.

The Maca sample was extracted in methanol at the ratio 1:30 (*w*/*v*) of Maca to methanol at room temperature for 12 h. The mixture was shocked with ultrasonic technique at room temperature for 30 min, then filtered. Barium acetate was added to the filtrate to precipitate the protein. MCE was obtained after concentrating the solution under vacuum.

The method of purification referred to Wang’s report with minor modification [36]. D301 macroporous anion resin was preliminarily used to purify concentrated MCE. When the sample solution was poured into the column filled with D301 macroporous anion resin, it was first washed with water and eluted with potassium nitrate solution. Then, the eluent was dried by rotary evaporation. Because glucosinolate is soluble in ethanol, and salt in eluent is not soluble in ethanol, salt can be removed from eluent after ethanol was added and the mixture was filtered. A small amount of ethanol was used to evaporate the sample under vacuum at 50 °C and to desalt after dissolution equilibrium. Ethanol was added to the filtered liquor (20:1 *v*/*v*) to form ethanol precipitate. The precipitate was dried under vacuum.

To obtain purified GLS, the above sample was further purified with acidic alumina column. The eluant was made up of 70% ethanol, deionized water, and 0.1 mol/L potassium nitrate. In addition, a small amount of methanol was added to the eluant to desalt. To obtain total GLS, 10 times the volume of ethanol was added to the sample. The mixture was placed at −20 °C overnight, then the precipitate was dried under vacuum at 50 °C.

### 3.3. Enzymatic Hydrolysis

A total of 25 UN thioglucosidase was added to pH 7.0 phosphate buffer solution. The solution was diluted to 1 U/mL and stored at −20 °C for further use. GLS dissolved in the buffer solution with the concentration of 10 mg/mL for further use.

The enzymatic hydrolysis method of glucosinolate was a little modified with reference to the method reported by Su et al. [37]. After 0.02 mmol EDTA and 0.2 mg ascorbic acid were mixed with the enzyme solution at the ratio 1:1 (*v*/*v*) of enzyme to substrate, the mixture was incubated in water at 40 °C for 4 h [24,25]. The mixture with 3 mL dichloromethane was extracted at room temperature for 2 h. ITCs were obtained from the lower extract after centrifugation at 6000 rpm.

### 3.4. Characterization

The UV absorption property of samples was recorded with a UV-8000S UV–Vis spectroscopy (Shanghai Precision Instrument Co., Ltd., Shanghai, China) in the wavelength rang of 150–300 nm.

The chemical structure of samples was determined by FT-IR absorption spectrometer (Bruker Daltonics, Bremen, Germany) in the wavenumber range of 4000 to 500 cm^−1^.

UPLC-MS was used to analyze purified GLS from Maca with modifications [38]. An UltiMate 3000 Series UPLC system consisting of a Thermo Fisher Scientific (Waltham, MA, USA) quaternary pump, thermostatic auto-sampler, and UV–Vis detector was used. An Acclaim™ RSLC 120C18 (2.1 mm × 50 mm, 2.2 μm) chromatographic column was supplied by Agilent Technologies (Burlington, MA, USA). The experimental parameters of UPLC were set as follows: the column temperature, 25 °C; UV detection wavelength, 200 nm; flow rate, 0.2 mL/min. The mobile phase consisted of 0.1% CF_3_COOH in deionized water (A) and 0.1% CF_3_COOH in methanol (B), respectively. The experimental parameters of gradient elution were set as follows: 0–7 min 98% A and 2% B; 7–20 min 88% A and 12% B.

Internal standard method was used for quantitative analysis of GLS from Maca with sinigrin as the internal standard [39]. GLS content was calculated according to the following Equation (1):

(1) $$\mathrm{Glucosinolate}\;\mathrm{content}\;(\mu g/\mathrm{mg})=\frac{A_{1}}{A_{0}}\cdot \frac{m_{1}}{m_{0}}$$

where *A*_1_ is peak area of each component, *A*_0_ is peak area of sinigrin, *m*_1_ is the mass of sinigrin, and *m*_0_ is the mass of sample.

A ratio of 1 mg/mL sinigrin was added to the sample for quantitative analysis comparing with the peak area.

Mass spectra were gained using a micrOTOF II time-of-flight mass spectrometer (Bruker Daltonics, Bremen, Germany). The experimental parameters of mass spectrometer were set as follows: flow rate of the sample solution, 3 μL/min; flow rate of nitrogen, 8 L/min; temperature of dry gas, 200 °C; voltage of capillary, 2.6 kV; voltage of end plate offset, 0.5 kV. MicrOTOF control software was used to acquire the data at the *m*/*z* range of 200–700 in negative ion mode.

ITCs were determined by GC-MS (ThermoFisher, Waltham, MA, USA) using a Thermo Scientific TraceGOLD TG-1701MS capillary column (30 m × 0.25 mm × 0.25 μm). The experimental parameters of GC-MS were set as follows: carrier gas, helium; constant flow rate, 1.5 mL/min; temperature of injection, 300 °C; injection volume, 10 μL; split ratio, 20:1; electron impact (EI) ion source temperature, 230 °C; interface temperature, 250 °C; quadrupole temperature, 150 °C; EI energy, 70 eV; scanning range of *m*/*z*, 50–240.

The temperature program of GC-MS was set as follows: 50 °C for 2 min, increased to 230 °C at a rate of 10 °C/min, holding for 5 min, and increased to 300 °C at a rate of 30 °C/min, holding for 15 min.

### 3.5. Antioxidant Activity In Vitro

DPPH radical scavenging assay: The antioxidant activity of total GLS and MCE was evaluated according to the method reported by Sun T. [40]. Total GLS and MCE were prepared into samples with a concentration of 10 mg/mL, and V_C_ with the same concentration was used as the positive control. A volume of 2.5 mL of DPPH radical solution was added to 2.5 mL of the sample solution at various concentrations. Then, the mixture was stood at room temperature for 30 min. Using ethanol as blank, the absorbance of sample was measured at 517 nm by UV–Vis absorption spectroscopy. DPPH radical scavenging capacity of the sample was calculated according to the following Equation (2):

(2) $$\mathrm{Scavenging}\;\mathrm{rate}\;\left(\%\right)=\left(1-\frac{A_{1}-A_{2}}{A_{0}}\right)\times 100$$

where *A*_0_ is the absorbance of the negative control (DPPH without sample solvent), *A*_1_ is the absorbance of the DPPH system with sample addition, and *A*_2_ is the absorbance of the sample background (samples together with ethanol).

ABTS radical scavenging assay: ABTS radical scavenging assay of total GLS and MCE was performed according to the method of Wang W. et al. with slight improvement [41]. A 2.45 mmol/L K_2_S_2_O_8_ solution and a 7 mmol/L ABTS radical solution were mixed and the mixture was stood overnight. The mixture was diluted with ethanol until the absorbance was about 0.70 ± 0.02 at 752 nm. Then, 0.5 mL of samples with different concentrations were mixed with 3.00 mL of diluted ABTS free radical stock solution. The above sample stood for 15 min. Using ethanol as blank, the absorbance of sample was measured at 752 nm. V_C_ with the same concentration was used as the positive control. ABTS radical scavenging activity was calculated according to the following Equation (3).

(3) $$\mathrm{Scavenging}\;\mathrm{rate}\;\left(\%\right)=\left(1-\frac{A_{1}-A_{2}}{A_{0}}\right)\times 100$$

where *A*_0_ is the absorbance of the negative control (ABTS without sample solvent), *A*_1_ is the absorption of the ABTS system with sample addition, and *A*_2_ is the absorption of the sample background (samples together with ethanol).

Reducing power assay: Reducing power of the sample was measured with reference to the method reported by Wang C.C. et al. [27]. A volume of 1.00 mL of sample was mixed with 2.5 mL of PBS (pH 6.6) and 2.5 mL of 1.00% K_3_[Fe(CN)_6_] solution, then shocked in a water bath at 50 °C for 20 min. The mixture was cooled to room temperature, and 2.5 mL of 10% CCl_3_COOH was added. The solution was centrifuged at 6000 rpm for 10 min. After adding 2.5 mL of deionized water and 0.5 mL of 0.1% FeCl_3_ solution to 2.50 mL of the supernatant, the mixture stood for 10 min. Optical density of the sample was measured at the wavelength of 700 nm. Using V_C_ as the positive control, reducing power was determined according to the above method. Because reducing power of the sample is directly proportional to optical density, the optical density of sample was used to evaluate reducing power.

### 3.6. Cytotoxic Assay

MTS was used to determine the inhibitory effect of samples on HL-60 leukemia, A549 lung cancer, SMMC-7721 liver cancer, MCF-7 breast cancer cells, and SW480 colon cancer cells [42]. These cell lines were obtained from ATCC (Manassas, VA, USA).

Cells were cultured in DMEM or RMPI-1640 medium (Biological Industries, Kibbutz Beit-Haemek, Israel) supplemented with 10% fetal bovine serum (Biological Industries) at 37 °C in a humidified atmosphere with 5% CO_2_. A ratio of 100 U/mL penicillin-streptomycin solution was used for sterilization. The cytotoxicity was evaluated by the 3-(4,5-dimethylthiazol-2-yl)-5-(3-carboxymethoxyphenyl)-2-(4-sulfophenyl)-2H-tetrazolium inner salt (MTS) (Promega, Madison, WI, USA) assay. A total of 100 μL per well and 3000–15,000 cells per well in 96 well plates were cultured for 12–24 h.

The sample (100 μg) was dissolved in 1 mL of pure DMSO. Due to the toxicity of DMSO, the content of DMSO in culture medium was determined by the type of cell line in cytotoxic assay. A volume of 200 μL/well of sample was incubated to initially screen at 37 °C for 48 h. Well–culture medium was removed from the adherent cells. A volume of 20 μL of MTS and 100 μL of culture medium were added to each well. A volume of 100 μL of culture medium was removed from the suspension cells. A volume of 20 μL of MTS was added to each well. Three blanks were made up of 20 μL of MTS and 100 μL of culture medium. After incubation for 2–4 h, the absorbance of each well was measured at 492 nm using a microplate reader (Bio-tek Multiskan FC, Vermont, USA). IC_50_ value of each compound was calculated by Cory’s report with cisplatin and taxol as positive controls [31]. Cell viability was calculated according to the following Equation (4):

(4) $$\mathrm{Cell}\;\mathrm{viability}\;\left(\%\right)=\frac{A_{1}}{A_{0}}\times 100\%$$

where *A*_1_ is the mean absorbance of well treated with the tested sample, and *A*_0_ is the mean absorbance of untreated cells.

### 3.7. Anti-Inflammatory Assay

RAW264.7 macrophages were plated into 96-well plates. Then, the macrophages were incubated with 1 μg/mL LPS and the sample. Nitric oxide synthase inhibitor (NOSI) was used as positive control, and the culture medium without sample was used as control group. After the cells were cultured overnight, the culture medium was used to detect the production of NO. The optical density was measured to detect nitrite (NO_2_^−^) at 570 nm wavelength by Griess method. To remove the effect of the sample on the cell toxicity, MTS was added to the remaining medium to detect the cell viability [43]. Anti-inflammatory activity was calculated according to the following Equation (5).

(5) $$\mathrm{Inhibition}\;\mathrm{rate}\;\mathrm{of}\;\mathrm{NO}\;\left(\%\right)=\frac{A_{1}-A_{0}}{A_{1}}\times 100\%$$

where *A*_1_ is optical density at 570 nm in non-drug treatment group, and *A*_0_ is optical density at 570 nm in control group.

### 3.8. Statistical Analysis

Statistical analysis of variance was calculated by software SPSS Statistics (Version 22.0, Chicago, IL, USA). Data were subjected to one-way analysis of variance (ANOVA), Ifollowed by multiple comparisons with least significant differences (LSD) test or Dunnett’s test as appropriate. Statistical significance was considered with *p* < 0.05. All the data were expressed as the mean ± SD (*n* = 3).

## 4. Conclusions

Crude extracts of GLS from fresh and dried Maca were purified by macroporous resin and acidic alumina column. Purified GLS were characterized by UV-Vis, FT-IR, and UPLC–ESI–MS. Purified samples contained three aromatic GLS including GTL, GLH, and GSB. The results show that this method can effectively remove most impurities and enrich GLS in Maca. GLS content in fresh Maca was more than that in dried Maca. BITC was obtained after enzymolysis of GLS. Bioactivities including antioxidation, antitumor, and anti-inflammation of total GLS and BITC were evaluated. Free radical scavenging ability of total GLS is weaker than that of MCE, while BITC had no antioxidant activity. Total GLS in Maca had no obvious antitumor and anti-inflammatory activities, while BITC had strong antitumor and anti-inflammatory activities (*p* < 0.05). Cell viability of 100 μg/mL BITC on five human tumor cells including leukemia HL-60, lung cancer A549, liver cancer SMMC-7721, breast cancer MCF-7, and colon cancer SW480 are 0.68%, 0.33%, 0.15%, 2.02%, and 1.99%, respectively. In addition, inhibition rate of NO production of BITC can reach 99.26% at 50 μg/mL, and 88.44% at 1.56 μg/mL. Due to its excellent anti-cancer and anti-inflammatory activities, BITC may be used as a promising antitumor and anti-inflammatory drug. However, several other crucial factors in terms of pharmacokinetics must be further studied for this phytomolecule to be considered as a potential drug candidate. It was further concluded that there was no correlation between the antioxidant, anti-cancer, and anti-inflammatory activities of total GLS and their enzymolysis product. A robust analysis method of GLS and bioactivity evaluation of other GLS and their enzymolysis products are currently in progress in our laboratory.

## Figures and Tables

**Table 1:** The content of benzyl glucosinolate (GTL), *m*-methoxybenzyl glucosinolate (GLH), *p*-hydroxybenzyl glucosinolate (GSB), and glucosinolates (GLS) in fresh Maca and dried Maca.

Glucosinolate	Content in Fresh Maca (mg/g)	Content in Dry Maca (mg/g)
GTL	24.31 ± 2.5	19.37 ± 0.67
GLH	3.72 ± 0.63	2.81 ± 0.31
GSB	6.09 ± 0.43	4.14 ± 0.39
GLS	34.12 ± 2.4	26.32 ± 1.4

**Table 2:** The effect of concentration of benzyl isothiocyanate (BITC) on cell viability (%).

Concentration (μg/mL)	Cell Viability (%)				
	HL-60	A549	SMMC-7721	MCF-7	SW480
0.16	88.31 ± 2.10	95.12 ± 0.90	86.22 ± 2.70	92.79 ± 4.70	88.50 ± 1.60
0.8	53.34 ± 2.53	55.29 ± 0.38	54.75 ± 0.66	71.24 ± 0.93	58.58 ± 1.30
4	0.89 ± 0.17	21.74 ± 2.07	7.54 ± 2.08	31.15 ± 1.72	24.57 ± 0.15
20	0.54 ± 0.14	3.75 ± 0.48	−0.34 ± 0.09	8.05 ± 0.74	25.26 ± 2.43

All data are represented as mean ± SD. *n* = 3.

**Table 3:** The IC_50_ values of BITC, paclitaxel, and cisplatin (DDP) on the five tumor cell lines, respectively.

Sample	IC_50_ ± SD (μg/mL)				
	HL-60	A549	SMMC-7721	MCF-7	SW480
BITC	0.886 ± 0.065 *	1.021 ± 0.032 *	0.941 ± 0.023 *	1.880 ± 0.088 *	1.200 ± 0.057 *
paclitaxel	<0.006	<0.006	0.033 ± 0.004	<0.006	<0.006
DDP	1.797 ± 0.318 *	3.669 ± 0.33 *	1.298 ± 0.306 *	6.669 ± 1.09 *	6.309 ± 0.98 *

All data are represented as mean ± SD. *n* = 3. * *p* < 0.05 compared to DDP group.

**Table 4:** Inhibition rate of NO production of nitric oxide synthase inhibitor (NOSI), GLS extracted from dried Maca (D-GLS), GLS extracted from fresh Maca (F-GLS), benzyl glucosinolate (GTL), and benzyl isothiocyanate (BITC).

Sample	Concentration (μg/mL)	Inhibition Rate of NO (%)
NOSI	12.41	58.07 ± 0.89
F-GLS	50	−10.73 ± 0.57
D-GLS	50	−10.13 ± 2.81
GTL	50	16.19 ± 2.53
BITC	50	99.26 ± 0.62 *
	1.56	88.44 ± 1.58 *

All data are represented as mean ± SD. *n* = 3. * p < 0.05 compared to nitric oxide synthase inhibitor.

## Data Availability

All relevant data can be found within the manuscript and its supporting materials.

## Supplementary Materials

The supporting information can be downloaded at: https://www.mdpi.com/article/10.3390/ijms232314756/s1.
