# Supplementary material for: Evaluation of the Biological Activity of Glucosinolates and Their Enzymolysis Products Obtained from Lepidium meyenii Walp. (Maca)
Source: Int J Mol Sci. 2022 Nov 25;23(23):14756. doi: 10.3390/ijms232314756 (PMC9740802; doi:10.3390/ijms232314756)
Supplement: Supplementary file 1 [file ijms-23-14756-s001.zip › ijms-1969969-supplementary.pdf]

Evaluation of the Biological Activity of Glucosinolates and Their Enzymolysis Products Obtained from *Lepidium meyenii* Walp. (Maca)

Suitong Yan[1], Jinchao Wei[2], Rui Chen[1, *]

[1] College of Chemistry and Chemical Engineering, Yunnan Normal University, Kunming 650500, China

[2] Institute of Chemistry, The Chinese Academy of Sciences, Beijing 100190, China

Corresponding author: Rui Chen

College of Chemistry and Chemical Engineering, Yunnan Normal University, Kunming 650500, China

E-mail: 4364@ynnu.edu.cn

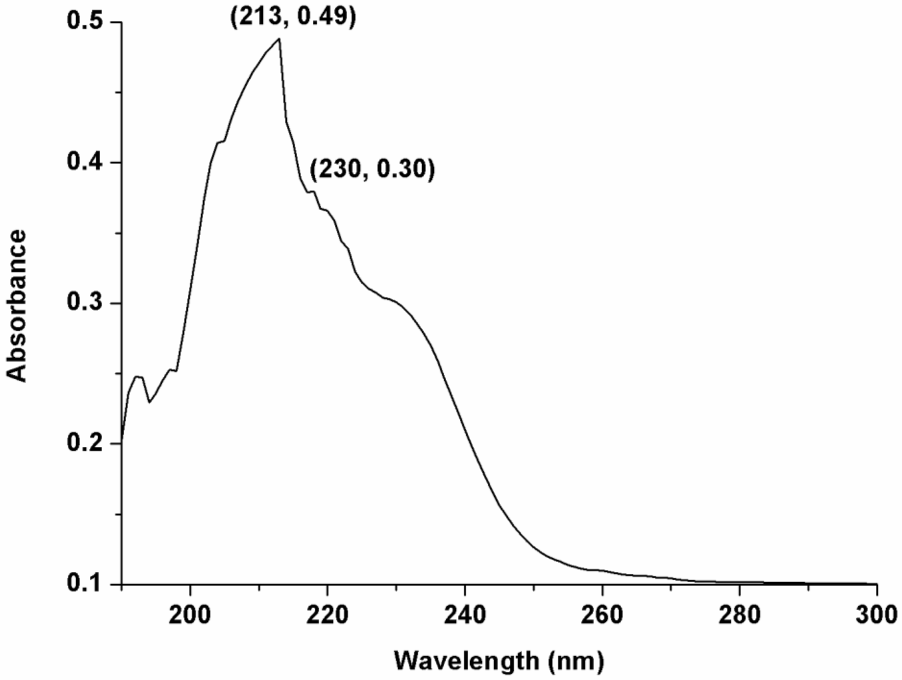

Figure S1 Ultraviolet absorption spectroscopy of glucosinolates.

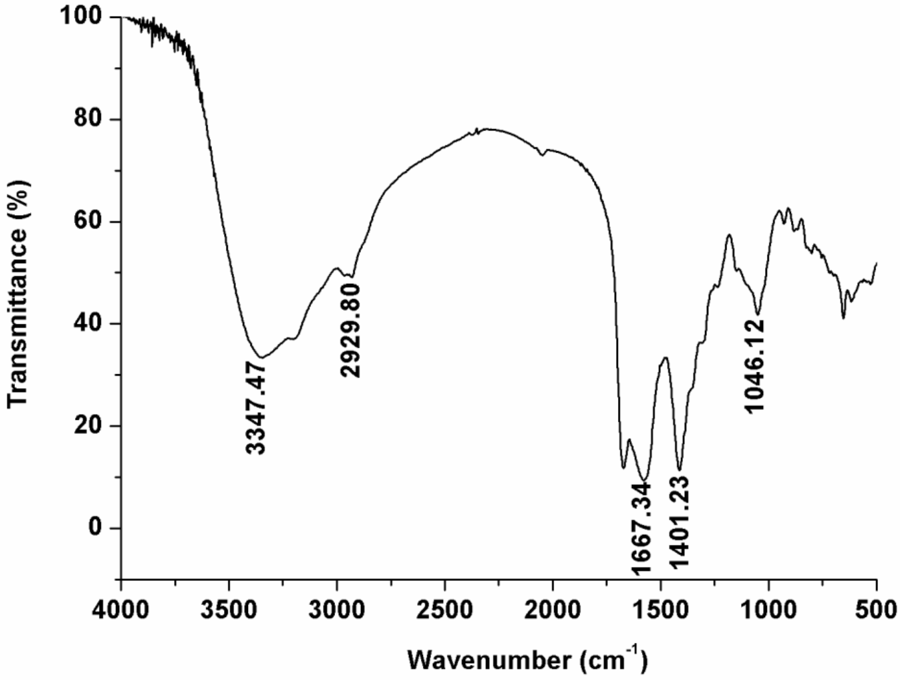

Figure S2 Fourier-transform infrared absorption spectroscopy of glucosinolates.

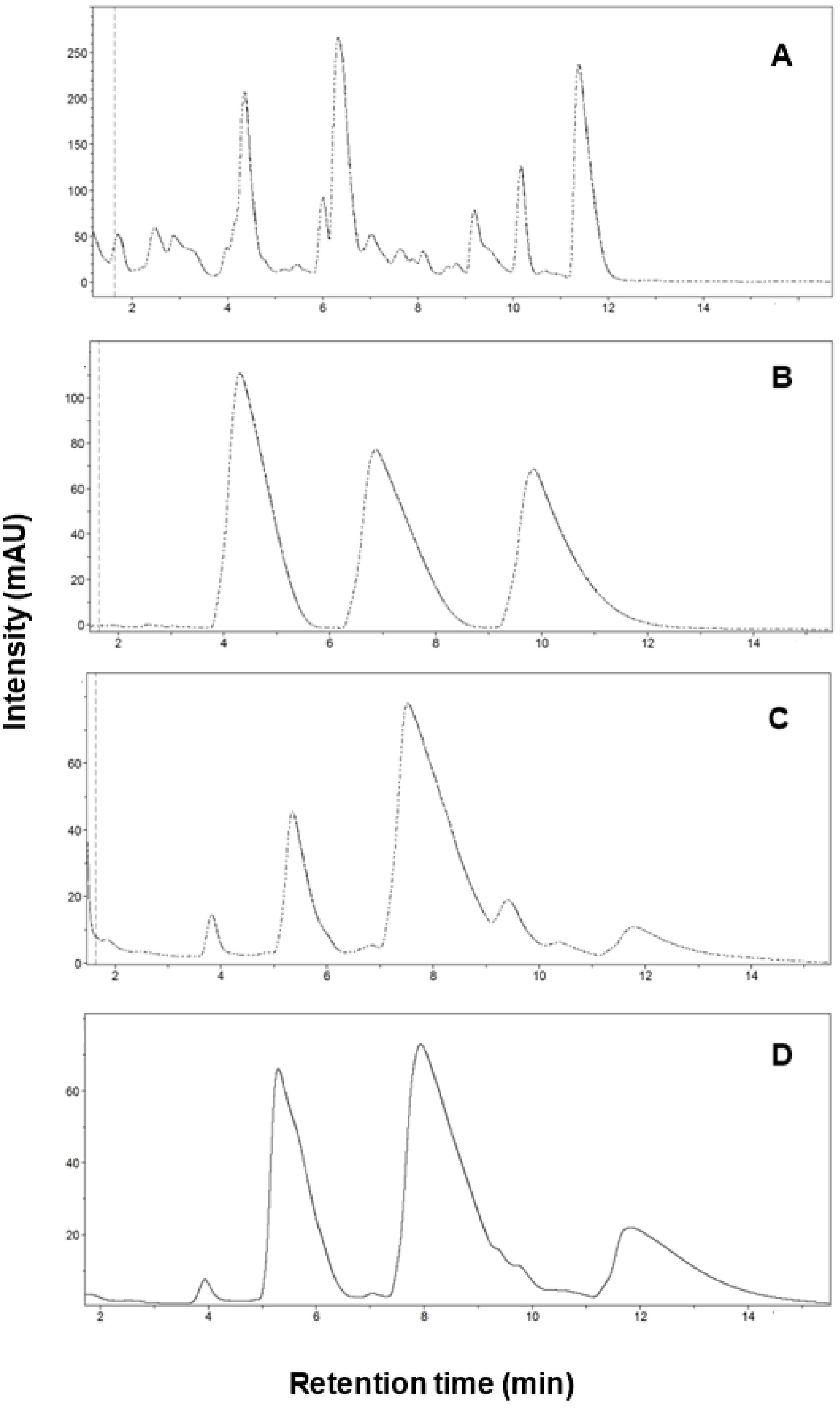

Figure S3 Ultra-high performance liquid chromatograms of Maca crude extract (A), standards (B), purified sample (C) and spiked sample (D), respectively.

Table S1 Characterization of*p*-hydroxybenzyl glucosinolate (GSB), benzyl glucosinolate (GTL) and *m*-methoxybenzyl glucosinolate (GLH) by ultra-high performance liquid chromatography-electrospray ionization-mass spectrometry

Sample	Chemical formula	Chemical structure	Ion type detected by MS	Measured m/z of ion	Theoretical m/z of ion	Relative error (ppm)
GSB	$C_{14}H_{18}KNO_{10}S_2$	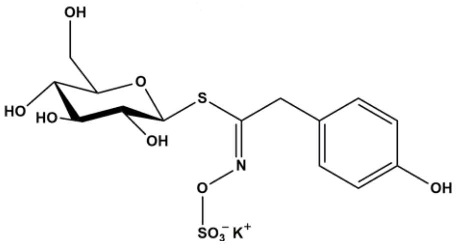	$[M-K]^-$	424.0389	424.0372	4.01
GTL	$C_{14}H_{18}KNO_9S_2$	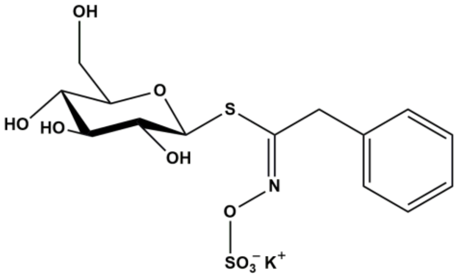	$[M-K]^-$	408.0443	408.0423	4.90
GLH	$C_{15}H_{20}KNO_{10}S_2$	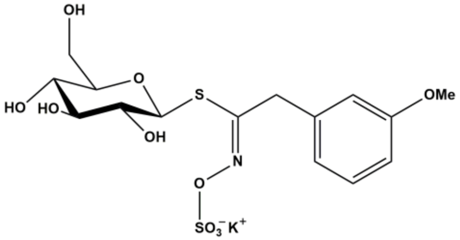	$[M-K]^-$	438.0548	438.0529	4.34

Table S2 The effects of glucosinolates extracted from dried Maca (D-GLS) and fresh Maca (F-GLS), benzyl glucosinolate (GTL), and benzyl isothiocyanate (BITC) at 100 μg/mL on growth inhibition of different tumor cell lines

Sample	Cell inhibition rate (%)				
	HL-60	A549	SMMC-7721	MCF-7	SW480
D-GLS	19.01±0.32	2.88±2.36	2.35±2.38	17.06±2.91	29.14±2.57
F-GLS	27.58±1.14	4.08±0.76	7.09±1.39	11.73±1.58	13.83±3.10
GTL	2.60±2.15	10.42±0.12	6.36±0.60	18.25±1.66	16.64±1.86
BITC	99.32±0.19	99.67±0.07	99.85±0.09	97.98±0.64	98.01±0.11
